# The impact of a peer-based education on fruits and vegetables intake among housewives

**DOI:** 10.1186/s41043-021-00278-3

**Published:** 2021-12-13

**Authors:** Roya Sheybani, Zahra Hosseini, Sayed Hossein Davoodi, Teamur Aghamolaei, Amin Ghanbarnejad

**Affiliations:** 1 Student Research Committee, Health School, Hormozgan University of Medical Sciences, Bandar Abbas, Iran; 2 Tobacco and Health Research Center, Hormozgan University of Medical Sciences, Bandar Abbas, Iran; 3 Department of Nutrition Research, National Nutrition and Food Technology Research Institute, Faculty of Nutrition Sciences and Food Technology, Shahid Beheshti University of Medical Sciences, Tehran, Iran; 4 Social Determinants in Health Promotion Research Center, Hormozgan Health Institute, Hormozgan University of Medical Sciences, Bandar Abbas, Iran

**Keywords:** Fruits and vegetables, Peer education, Housewives

## Abstract

**Background:**

Evidence indicates the lower intake of fruits and vegetables than the recommended daily amount. Study aimed at determining the effects of peer education intervention on the consumption of fruits and vegetable in housewives.

**Methods:**

A quasi-experimental was conducted with 130 housewives referring to health care centers in Bandar Abbas, Iran. Sixty-five subjects were recruited in each of the intervention and the control groups. Intervention group were divided into three subgroups each receiving a seven-sessions educational programs (lecturing and group discussion) through peers about the importance of benefits of fruits and vegetables consumption. Participants were followed for two months. Data were collected using a questionnaire in two stages of pre- and post-intervention. Differences in the outcome before and after the intervention were tested using *T*-test and paired *T*-test.

**Results:**

The daily servings of fruits and vegetables in the intervention group increased from 1.73 to 4.20 and in the control group from 1.96 to 2.16; a statistically significant difference was also observed between the groups (*P* < 0.001). After the intervention benefits and self-efficacy of fruits and vegetables consumption significantly increased and perceived barriers of fruits and vegetables consumption significantly decreased in the intervention group (*P* < 0.001).

**Conclusion:**

Peer education improves benefits and self-efficacy, reduces barriers, and increases the daily servings of fruits and vegetables in housewives.

## Background

Fruits and vegetables are the main part of a healthy diet that has multitude positive impacts on health and reduction of the risk of chronic illnesses [1]_._ According to a study performed on Swedish females aged 40–76 years, high amount of fruits and vegetables consumption reduced the risk of renal cell carcinoma [2]. Also, high intake of fruits and vegetables can reduce the risk of breast cancer [3]. Fruits and vegetables are rich in antioxidants, vitamins, soluble fibers, and other nutrients [4], which prevent obesity and overweight [5].

Despite the benefits of fruits and vegetables in the improvement of health and reduction in the burden of chronic diseases, at least five daily servings of fruits and vegetables is not common among different populations [6]. According to the results of different studies, only less than one-third of adults in the United States eat the recommended daily servings of fruits and vegetables [7]. Studies in Iran also show lower amount of daily fruits and vegetables intake than recommended. Promotion of healthy nutrition behaviors is fruitful if the factors affecting health behaviors are identified [8].

Females constitute half of the population of each country, which have to be educated in health and nutrition in order to affect family health. According to the specific cultural and traditional features of Iran, females can more easily share health issues.

The peer education is one of the health education approaches to change the behavior. Peer education can develop knowledge and skills through interpersonal active support and collaborative approach. Forming peer groups in a particular social class leads to a constructive dialogue between them through which a certain function is selected and finally the behavior of the group members is changed. Peer educators are eligible to hold or contribute to the implementation of educational programs. Such people are usually accepted by the intervention group and are influential among them, and by passing skill training courses, they can play the role of an educator [9].

Due to the importance and benefits of fruits and vegetables intake for the health of the community and prevention of chronic diseases, and also owing to the importance of females nutritional health as part of the community and their roles as mothers, and the impact of females on the health of children and other family members, the current study aimed at determining the effect of peer education intervention on the benefits, barriers, self-efficacy, and intake of fruits and vegetables in housewives.

## Methods

### Study population and sampling

The current quasi-experimental study was conducted in 2018 on housewives were referring to health centers in Bandar Abbas, the capital of Hormozgan province in southern Iran. Out of the 22 centers, 2 centers were randomly selected. these centers are located in relatively homogeneous region with socioeconomic status Medium. The sample size was 65 in each of the intervention and control groups. Sampling in each group was done by simple random sampling method based on the family file number in each health center. The inclusion criteria were being married, reading and writing ability, and willingness to participate in the study. The exclusion criteria were being an emigrate, no attending training sessions regularly and incomplete response to the questionnaire in pre-test or post-test. The far distance between the two health centers made the communication between the participants impossible. participants were selected through simple random sampling based on the family file number at the health centers. The participants were informed that participation in the study was voluntary and they had the right to withdraw at any time during the study process. All participants were given consent forms to sign when they voluntarily opted to be part of this study.

### Data collection

The data were collected using a questionnaire at two stages (before and two months after the intervention). The questionnaires included 3 parts: the demographic characteristics of the study participants; included age, education level. We designed a questionnaire to examine the effect of percieved benefits, percieved barriers, and self-efficacy of fruits and vegetables intake on house wives. This questionnaire consisted of 25 questions, includes (percieved benefits: 10, percieved barriers: 8 and self-efficacy: 7) for fruits and the same items were considered for vegetables consumption that way instead of fruit, vegetable was written. To measure percieved benefits, percieved barriers, the Likert scale had five options from strongly agree to strongly disagree and for self efficacy from very easy to so hard (Table 1). The questionnaire also had an item to measure the daily servings of fruits and vegetables. Before scoring this item, the definition and examples for better understanding were provided for “daily serving” of fruits and vegetable. Validity and reliability of the questionnaire were confirmed in previous studies [10]. The reliability of the questionnaire in this study was 0.71, 0.69 and 0.75 for each of the constructs of perceived benefits, perceived barriers and self-efficacy, respectively. To determine the validity, a test–retest was used and the questionnaire was given to 18 participants apart two weeks and was confirmed with a correlation coefficient of 0.71.

**Table 1:** Perceived benefits, perceived barriers and self-efficacy items of housewives in related to fruit consumption (Intervention Group)

Factors	Cronbach's alpha	Items	Mean (before)	Mean (after)	Range
Percieved benefits	0.71	Fruit is good for health	4.00 (0.90)	4.81 (0.39)	10–50
		Fruit can be a substitute for unhealthy foods	3.58 (1.22)	4.61 (0.49)	
		Fruit intake can help maintain a healthy body weight	3.06 (1.36)	4.70 (0.45)	
		Fruit intake can prevent the disease	4.03 (1.18)	4.73 (0.44)	
		Fruit consumption makes you feel freshness	3.93 (0.96)	4.67 (0.50)	
		The consumption of fruit causes a diets variety	4.24 (0.72)	4.67 (0.47)	
		The consumption of fruit causes a person to lose his extra weight	3.63 (1.31)	4.60 (0.60)	
		Fruit consumption provides the vitamins and minerals necessary for the body	4.20 (0.71)	4.81 (0.39)	
		Fruits contain fiber and antioxidants	4.20 (0.77)	4.78 (0.41)	
		Fruit consumption somewhat provides water for the body	2.50 (1.23)	4.70 (0.45)	
Percieved barriers	0.69	I do not have access to fruit	2.33 (1.31)	2.38 (0.89)	8–40
		Finding delicious fruit is difficult	2.60 (1.35)	2.44 (0.90)	
		I do not have the facilities for keeping fruit	1.60 (0.70)	2.09 (0.70)	
		Fruits are not tasty	3.84 (1.41)	2.26 (0.77)	
		Fruits are expensive	4.07 (0.85)	3.23 (0.91)	
		Fruits quickly deteriorate	3.18 (1.36)	3.07 (0.97)	
		I do not have enough time to buy fruit	2.40 (1.11)	2.92 (0.97)	
		Fruits are contaminated with chemicals due to spraying pesticide	4.12 (0.81)	3.61 (o.76)	
Self-efficacy	0.75	Is it hard or easy for you to eat fruit in the following situations:			7–35
		When daily working	3.10 (1.13)	4.20 (0.66)	
		When you do not have enough time	1.7890.73)	2.75 (0.66)	
		When you feel tired	2.47 (1.61)	2.61 (0.72)	
		When you are sick	2.41 (1.44)	2.49 (0.75)	
		When the fruit you like is not available	1.76 (0.72)	2.50 (0.85)	
		When you are not hungry	2.33 (1.22)	2.86 (0.93)	
		When you do not have the desire to eat fruit	2.21 (1.16)	2.87 (0.87)	

### Educational intervention

First, five females of the study population were recruited as trainers based on the inclusion criteria; i.e., acceptability, permissiveness, and appropriate communication skills. They received the necessary training in order to transfer educational materials to other females. Then they were trained in order to further train other female subjects. They were also familiarized with lecturing methods, group discussion, and question and answer. Accordingly, the trainers educated their peers in the intervention group regarding the role and importance of fruits and vegetables intake via different methods such as lecturing, group discussions, question and answer sessions, creating a group chat on Telegram, and distribution of a pamphlet. The females in the intervention group were divided into three subgroups each receiving seven-session 1-h educational for 2 months. Lecturing and group discussion programs held by the educators in health centers, in a room with suitable educational facilities such as video projector and whiteboard. In order to further engage with females, the question and answer method was also used. Also, a group chat was created on Telegram in order to share information about the importance and role of fruits and vegetables intake and resolving their confusions. The training sessions continued in the intervention group for one month, during which trainers were involved with the research team and received the necessary guidance. Two months after the completion of the intervention as the second stage of the study, the questionnaires were distributed among the participants and were collected by visiting their homes.

### Data analysis

Data were analyzed with SPSS version 22 using *T*-test and paired *T*-test for intragroup and intergroup comparisons, respectively; *P* < 0.05 was considered as the level of significance.

## Results

Totally 130 housewives participated in the current study, of which 65 were in the intervention group and the other 65 in the control group. After the follow-up and data collection, all the selected subjects were enrolled in the study. The mean age of the females in the intervention and control groups was 35.3 ± 2.6 and 38.9 ± 6.9 years, respectively. In both groups, the majority of housewives had high school education. There was no significant difference between the intervention and control groups in terms of demographic variables.

Before the intervention, there was no significant difference between the groups in terms of percieved benefits, percieved barriers, and self-efficacy toward fruits intake, as well as percieved benefits, percieved barriers, and self-efficacy toward vegetables intake and the daily servings of fruits and vegetables.

**Fig. 1:**
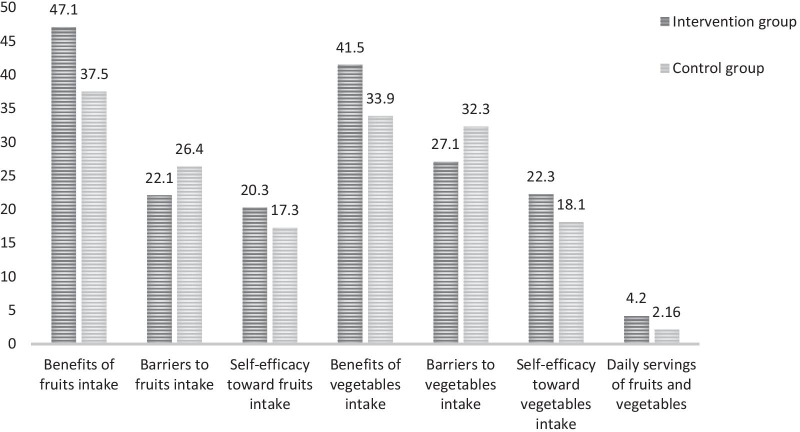
Comparison of the post-intervention scores of the intervention and control groups in terms of the studied variables

After the intervention, significant differences were observed between the groups (*P* < 0.001), the benefits and self-efficacy toward eating fruits, the benefits and self-efficacy toward vegetables intake and the daily servings of fruits and vegetables in the intervention group were higher than those of the control group, and in contrast, barriers to fruits and vegetables intake in the intervention group were lower than those of the control group (Fig. 1).

**Fig. 2:**
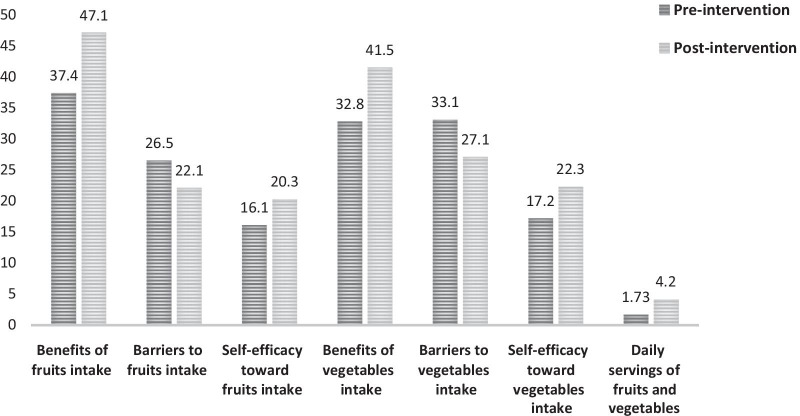
Comparison of the studied variables in the intervention group before and after the education intervention

A significant increase was observed in the scores of benefits and self-efficacy toward fruits intake, the benefits and self-efficacy toward vegetables intake, and the daily servings of fruits and vegetables, and a significant decrease in the barriers to fruits and vegetables intake in the intervention group, compared with pre-intervention scores (Fig. 2).

The pre- and post-intervention scores of the control group were compared in terms of benefits, barriers, and self-efficacy toward fruits intake, as well as the benefits, barriers, and self-efficacy toward fruits and vegetables intake and daily servings of fruit and vegetables (Table 2). According to the obtained results, there were no significant difference in any of the studied variables, except the self-efficacy toward vegetables and the daily servings of fruit and vegetables.

**Table 2:** Comparison of the studied variables in the control/intervention group before and after the intervention

Variable	Group	Pre-intervention	Post-intervention	*P* value**
		Mean ± SD	Mean ± SD	
Benefits of fruits intake	Control	36.5 ± 5.1	37.5 ± 4.5	0.19
	Intervention	37.4 ± 4.6	47.1 ± 2.7	< 0.001
	*P* value*	0.30	< 0.001	
Barriers to fruits intake	Control	26.2 ± 4.2	26.4 ± 5.7	0.79
	Intervention	26.5 ± 2.9	22.1 ± 1.4	< 0.001
	*P* value*	0.56	< 0.001	
Self-efficacy toward fruits intake	Control	16.9 ± 3.5	17.3 ± 3.2	0.45
	Intervention	16.1 ± 3.5	20.3 ± 3.5	< 0.001
	*P* value*	0.2	< 0.001	
Benefits of vegetables intake	Control	32.6 ± 3.9	33.9 ± 3.9	0.09
	Intervention	32.8 ± 3.4	41.5	< 0.001
	*P* value*	0.81	< 0.001	
Barriers to vegetables intake	Control	33.6 ± 3.6	32.3 ± 6.5	0.16
	Intervention	33.1 ± 3.5	27.1	< 0.001
	*P* value*	0.38	< 0.001	
Self-efficacy toward vegetables intake	Control	17.1 ± 3.3	18.1 ± 3.9	0.04
	Intervention	17.2 ± 3.1	22.3	< 0.001
	*P* value*	0.95	< 0.001	
Daily servings of fruits and vegetables	Control	1.98 ± 0.80	2.16 ± 0.76	0.03
	Intervention	1.73 ± 0.77	4.2 ± 0.81	< 0.001
	*P* value*	0.07	< 0.001	

*Independent sample *T*-test

**Paired sample *T*-test

## Discussion

This study examined the effect of peer education intervention on the benefits, barriers, self-efficacy, and daily servings of fruits and vegetables in housewives in Iran. Our results showed that a significant increase in benefits and self-efficacy and decrease in barriers to eating fruits in the intervention group compared with those of the control group after intervention. Similar findings were reported previously [11–15]. In Peyman's study the scores of benefits and perceived self-efficacy increased significantly after four sessions of healthy nutrition training [11]. In the study by Alizadeh, perceived barriers to nutritional behaviors decreased after education [12]. Di Noia and Thompson also reported that nutrition education programs can reduce perceived barriers [13].

After education in intervention group perceived the score of benefits and self-efficacy for all items increased. In perceived barriers scores of all items have been reduced other than: “I do not have access to fruit,” “I do not have the facilities for keeping fruit,” I “do not have enough time to buy fruit” The reason is the fruit is expensive. even if the person wants to buy fruits and vegetables from stores that are cheap may be do not have the necessary facilities to keep them. Because these stores sell a lot of fruit for example, they sell a basket of fruit, not a kilo of fruit. Also, the number of these stores is low and is probably far from them.

Also, the post-intervention scores showed that the benefits and self-efficacy toward eating vegetables were higher and the barriers to vegetables intake were lower in the intervention group than the control group. Findings were in line with those of Prochaska and Di Noja [16] in terms of the positive effects of education on the perceived benefits of vegetables intake, but inconsistent with the results of the study de Vet et al. [17]. The differences among the findings can be attributed to differences in the type and duration of the intervention and the educational content used in the studies. The current study results were consistent with those of Mainbolagh et al. [18] and Farvid et al. [19] in terms of the perceived barriers to vegetables intake. In the study by Vasalow, holding educational intervention increased the perceived self-efficacy toward nutritional behaviors and fruits and vegetables intake, which were consistent with the results of the current study [20]. Self-efficacy can maintain and improve health promotion behaviors [21]. In the study by Davoodi et al., the transition from the pre-thinking stage to the maintenance and preservation of behaviors associated with fruits and vegetables intake had a significant relationship with participants’ increased self-efficacy [8].

After education in intervention group the score of perceived benefits for all items increased. In perceived barriers scores of all items have been reduced other than: “vegetables are expensive” being expensive is a barrier to the consumption of vegetables before and after the intervention, too. The score of self-efficacy for all items increased other than: “When you are sick” Which can be due to physical and psychological conditions during illness.

After the peer education intervention, a significant increase was observed in the daily servings of fruits and vegetables in housewives in both the intervention and control groups, but the increase in the intervention group was significantly higher than that of the control group. Several studies promoted the nutritional behaviors of participants using educational programs, The control group did not receive the training of the intervention group but they had the routine training of the centers. This could be reason for increased consumption of fruits and vegetables in the control group [22].

The results of the study by Abbasian, evaluating the effect of an educational intervention on fruits and vegetables intake, showed that eating fruits and vegetables significantly increased after the intervention in the intervention group than the control group [23]. In the study by Green et al., a 24-month educational intervention was held to change the fruits and vegetables intake behavior in adults, and the results indicated a significant increase in the average daily servings of fruits and vegetables [24]. Stepto et al. [25] also showed that using the intervention program, the daily servings of fruits and vegetables significantly increased that was consistent with the current study findings. However, de Vet did not show the effect of educational intervention on fruits intake; in addition, the study by Park et al. [26] also showed that educational intervention could increase the vegetables intake, but had no impact on eating more fruits.

## Conclusion

Peer education can improve benefits and self-efficacy, reduce barriers, and increase the daily servings of fruits and vegetables in housewives. Therefore, it is recommended that such educational approaches should be employed to increase fruits and vegetables intake in housewives.

## Limitation

One of the limitations was the self-reported data collection, which can increase the probability of error due to the possibility of untrue reports. Another limitation was the lack of long-term follow-up of the participants, which is suggested to be considered in future studies.

## Data Availability

The datasets used and analyzed during the current study are available from the corresponding author on reasonable request.

## Abbreviation

SPSS: Statistical Package for the Social Sciences

## References

[1] Kimmons J, Gillespie C, Seymour J, Serdula M, Blanck HM (2009). Fruit and vegetable intake among adolescents and adults in the United States: percentage meeting individualized recommendations. Medscape J Med.

[2] Schoenaker DA, Soedamah-Muthu SS, Mishra GD (2014). The association between dietary factors and gestational hypertension and pre-eclampsia: a systematic review and meta-analysis of observational studies. BMC Med.

[3] Sangrajrang S, Chaiwerawattana A, Ploysawang P, Nooklang K, Jamsri P, Somharnwong S (2013). Obesity, diet and physical inactivity and risk of breast cancer in Thai women. Asian Pac J Cancer Prev.

[4] Krolner R, Rasmussen M, Brug J, Klepp KI, Wind M, Due P (2011). Determinants of fruit and vegetable consumption among children and adolescents: a review of the literature. Part II: qualitative studies. Int J Behav Nutr Phys Act.

[5] Gross SM, Davenport Pollock E, Braun B (2010). Family influence: key to fruit and vegetable consumption among fourth- and fifth-grade students. J Nutr Educ Behav.

[6] Conner TS, Brookie KL, Carr AC, Mainvil LA, Vissers MCM (2017). Let them eat fruit! The effect of fruit and vegetable consumption on psychological well-being in young adults: a randomized controlled trial. PLoS ONE.

[7] Di Noia J, Contento IR (2010). Fruit and vegetable availability enables adolescent consumption that exceeds national average. Nutr Res.

[8] Davoodi SH, Hosseini Z, Aghamolaei T (2017). Fruit and vegetable consumption in high school students in Bandar Abbas, Iran: an application of the trans-theoretical model. Arch Iran Med.

[9] Aghamolaei S, Rahmani T, Zare M, Ghanbarnejad A (2014). Effect of peer education on safety behaviors among workers of renovation of structures and machines shop in Bandar Abbas Oil Refinery Company. Iran J Health Educ Health Promot.

[10] Agah B, Aghamolaei T, Alizadeh A, Rafati S, Hosseini F (2015). Consumption of fruits and vegetables based on constructs of transtheoretical model in women referred to health centers of Bandar Abbas. J Prev Med.

[11] Peyman N, Heidarnia A, Ghofranipour F, Kazemnejad A, Khodaee GH, Shokravi FA. The relationship between perceived self-efficacy and contraceptive behaviors among Iranian women referring to health centers in Mashad in order to decrease unwanted pregnancies. J Reprod Infertil. 2007;8(1).

[12] Alizadeh Siuki H, Jadgal K, Shamaeian Razavi N, Zareban I, Heshmati H, Saghi N (2015). Effects of health education based on health belief model on nutrition behaviors of primary school students in Torbat e Heydariyeh city in 2012. Journal of Health.

[13] Di Noia J, Thompson D (2012). Processes of change for increasing fruit and vegetable consumption among economically disadvantaged African American adolescents. Eat Behav.

[14] Hildebrand DA, Betts NM (2009). Assessment of stage of change, decisional balance, self-efficacy, and use of processes of change of low-income parents for increasing servings of fruits and vegetables to preschool-aged children. J Nutr Educ Behav.

[15] Van Duyn MAS, Kristal AR, Dodd K, Campbell MK, Subar AF, Stables G (2001). Association of awareness, intrapersonal and interpersonal factors, and stage of dietary change with fruit and vegetable consumption: a national survey. Am J Health Promot.

[16] Di Noia J, Prochaska JO (2010). Dietary stages of change and decisional balance: a meta-analytic review. Am J Health Behav.

[17] de Vet E, de Nooijer J, de Vries NK, Brug J (2007). Testing the transtheoretical model for fruit intake: comparing web-based tailored stage-matched and stage-mismatched feedback. Health Educ Res.

[18] Mainbolagh BL, Rakhshani F, Zareban I, Montazerifar F, Sivaki HA, Parvizi Z (2012). The effect of peer education based on health belief model on nutrition behaviors in primary school boys. J Res Health.

[19] Farvid MS, Rabiee S, Homayoni F, Rashidkhani B, Arian V (2010). Determinants of fruit and vegetable consumption in type 2 diabetics in Tehran. Iran J Endocrinol Metab.

[20] Vassallo M, Saba A, Arvola A, Dean M, Messina F, Winkelmann M (2009). Willingness to use functional breads. Applying the Health Belief Model across four European countries. Appetite.

[21] Bandura A (1977). Self-efficacy: toward a unifying theory of behavioral change. Psychol Rev.

[22] Hosseini Z, Gharghani ZG, Mansoori A, Aghamolaei T, Nasrabadi MM (2015). Application of the theory of reasoned action to promoting breakfast consumption. Med J Islam Repub Iran.

[23] Abbasian F, Omidvar N, Bondarianzadeh D, Rashidkhani B, Shakibazadeh E, Hashemi B (2012). Effect of a school-based intervention based on social cognitive theory on fruit and vegetable consumption in middle school students in Tehran. Journal of hayat.

[24] Greene GW, Fey-Yensan N, Padula C, Rossi SR, Rossi JS, Clark PG (2008). Change in fruit and vegetable intake over 24 months in older adults: results of the SENIOR project intervention. Gerontologist.

[25] Steptoe A, Perkins-Porras L, McKay C, Rink E, Hilton S, Cappuccio FP (2003). Behavioural counselling to increase consumption of fruit and vegetables in low income adults: randomised trial. BMJ.

[26] Park A, Nitzke S, Kritsch K, Kattelmann K, White A, Boeckner L (2008). Internet-based interventions have potential to affect short-term mediators and indicators of dietary behavior of young adults. J Nutr Educ Behav.

